# Cisternotomy and Liliequist’s Membrane Fenestration as a Surgical Treatment for Idiopathic Intracranial Hypertension (Pseudotumor Cerebri): A Case Report

**DOI:** 10.7759/cureus.31363

**Published:** 2022-11-11

**Authors:** Akram M Eraky, Randall Treffy, Hirad S Hedayat

**Affiliations:** 1 Department of Neurosurgery, Medical College of Wisconsin, Milwaukee, USA

**Keywords:** liliequist’s membrane, lamina terminalis, cerebrospinal fluid (csf), glymphatic system, idiopathic intracranial hypertension (iih), pseudotumor cerebri syndrome (ptcs), cisternostomy, cisternotomy, subarachnoid cisterns

## Abstract

Subarachnoid basal cistern opening (cisternotomy) is used during many microsurgical operations to relax the brain by removing or diverting cerebrospinal fluid (CSF). Recently, cisternotomy has been used in patients with traumatic brain injury to improve outcomes due to its ability to decrease intracranial pressure (ICP) and brain edema by diverting CSF. Theoretically, another condition that can benefit from cisternotomy is idiopathic intracranial hypertension (IIH) as it presents with manifestations of increased ICP, such as headache, vomiting, and papilledema. Here, we discuss the case of a 39-year-old woman with IIH who presented with headache, nausea, and papilledema in the setting of maximally tolerated medical management after five months of shunt removal due to infection. The patient did not want to proceed with the replacement of her shunt and therefore underwent a right eyebrow craniotomy for cisternotomy, lamina terminals fenestration, and Liliequist’s membrane opening. Postoperatively, her symptoms improved completely. She was off acetazolamide altogether at the three-month follow-up and no longer had pseudotumor cerebri headaches. This case report demonstrates the use of cisternotomy to relieve the manifestations of increased ICP and its potential as a surgical option for patients with IIH.

## Introduction

Idiopathic intracranial hypertension (IIH) presents with manifestations of increased intracranial pressure (ICP), such as headache, pulsatile tinnitus, diplopia, vomiting, and papilledema [1]. Neuroimaging is required to diagnose IIH to exclude other causes of increased ICP, such as tumors. On magnetic resonance imaging (MRI), patients with IIH present with normal brain parenchyma and ventricles without hydrocephalus, mass lesions, or abnormal meningeal enhancement [1,2]. Elevated lumbar puncture opening pressure and symptom relief after lumbar puncture with normal cerebrospinal fluid (CSF) composition are characteristics of pseudotumor cerebri [1,2]. Traditional treatment for patients with IIH includes weight loss and carbonic anhydrase inhibitors [3,4]. Surgical management of cases refractory to medical therapy involves the placement of a CSF shunt [5,6]. Theoretically, another potential treatment of IIH is subarachnoid basal cistern opening (cisternotomy). Cisternotomy is used during many microsurgical operations to relax the brain by removing or diverting CSF [7]. Recent developments indicate that CSF from the ventricles does not communicate with the parenchyma but rather CSF from the cisterns communicates with the parenchyma through the Virchow-Robin spaces via the glymphatic system [8,9]. Based upon this, the release of CSF via cisternotomy is purported to increase the removal of interstitial fluid and CSF. A review of the literature did not reveal any similar cases. This case is considered the first reported case of IIH treated with cisternotomy.

## Case presentation

**Figure 1:**
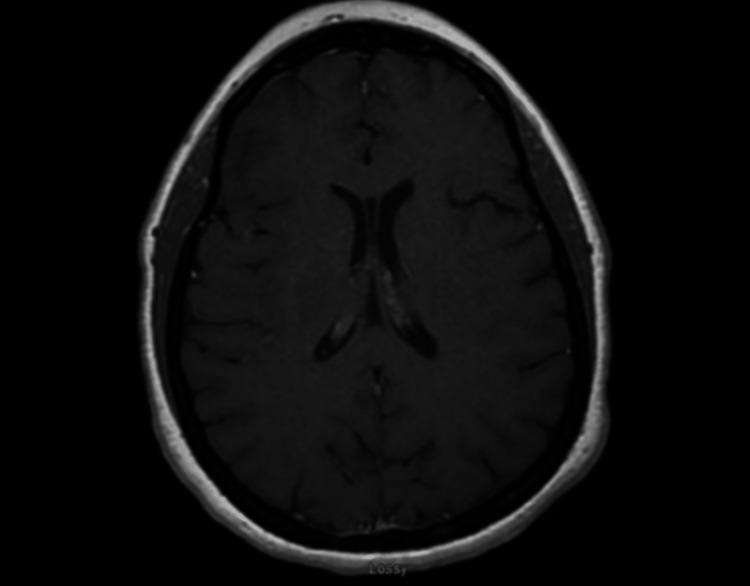
Preoperative T1 axial magnetic resonance imaging of the brain. Normal brain parenchyma and ventricles without hydrocephalus or mass lesions.

A 39-year-old woman presented with headaches and nausea and was diagnosed with IIH in the setting of maximally tolerated medical management. She did not have any other comorbidities. Her physical examination was unremarkable except for grade 1 papilledema. During hospitalization, she underwent imaging showing normal brain parenchyma and ventricles without hydrocephalus or mass lesions on MRI (Figure 1). Furthermore, MRI or MRI venography did not show venous sinus stenosis. The patient’s symptoms greatly improved with a lumbar drain trial; therefore, a ventriculoperitoneal shunt was placed.

**Figure 2:**
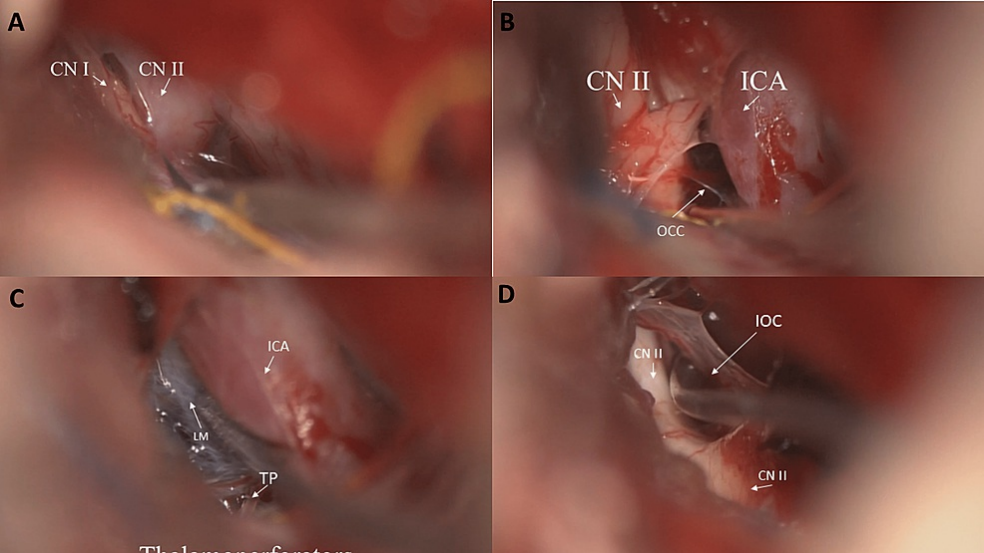
Cisternotomy through a right eyebrow craniotomy. (A) The view shows the olfactory nerve (CN I) and optic nerve (CN II). (B) The view shows the optico-carotid chiasm (OCC) opening, optic nerve (CN II), and internal carotid artery (ICA). (C) The view shows the OCC, thalamoperforators (TP), and Liliequist’s membrane (LM) between ICA and CN II. (D) The view shows the opening of the interoptic cistern (IOC).

Unfortunately, five months later, the patient’s shunt had to be completely removed due to infection, and acetazolamide was restarted. One month later, she presented with three weeks of progressive headaches and nausea. Headaches improved after a large-volume lumbar puncture showing an opening pressure of 27cmH_2_O. CSF labs were normal. The patient declined the replacement of her shunt as the next step in management and underwent a right eyebrow craniotomy for cisternotomy, lamina terminals fenestration, and Liliequist’s membrane opening (Figure 2).

**Figure 3:**
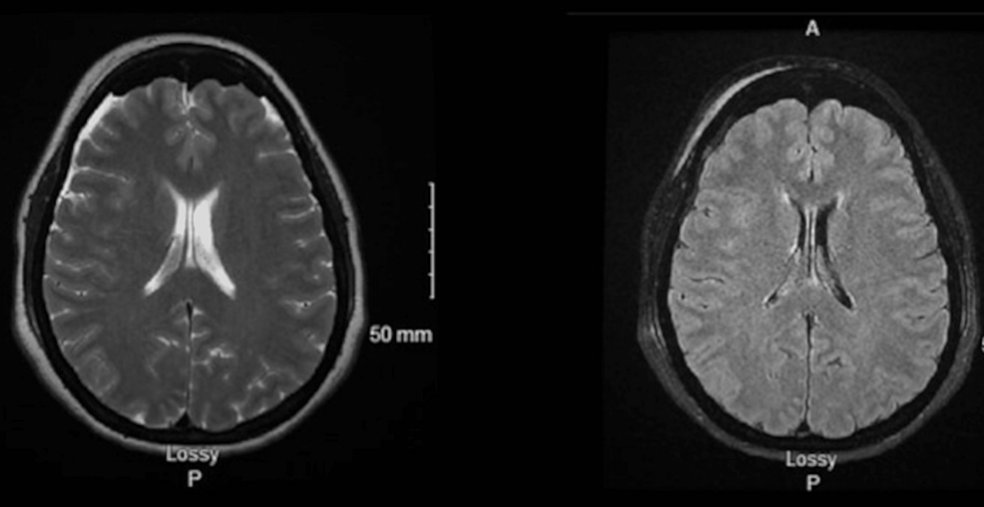
Axial magnetic resonance imaging of the brain. Normal brain parenchyma and ventricles without hydrocephalus or mass lesions.

During the surgery, the arachnoid was found to be exceptionally thick and required nearly exclusive sharp dissection (Figure 2). There were no postoperative complications, and the patient experienced complete resolution of her symptoms. She was off acetazolamide altogether at the three-month follow-up and no longer had IIH headaches. Postoperative MRI showed normal brain parenchyma and ventricles without hydrocephalus or mass lesions (Figure 3). Neuro-ophthalmology visit demonstrated stable grade 1 papilledema.

## Discussion

During many microsurgical operations, cisternotomy is used to relax the brain by diverting CSF. Recent developments indicate that CSF from the cisterns communicates with the brain parenchyma through the Virchow-Robin spaces via the glymphatic system [8,9]. Allowing CSF drainage through the cisterns by surgically opening them to atmospheric pressure may stimulate CSF and interstitial fluid drainage through the Virchow-Robin spaces, subsequently decreasing ICP [10]. Many previous clinical studies have shown that cisternotomy with decompressive craniotomy (DC) is associated with better outcomes when compared to DC alone in patients with traumatic brain injury [10,11]. Theoretically, other causes of increased ICP, such as IIH, can benefit from cisternotomy. In our case report, the patient’s symptoms improved after cisternotomy with Liliequist’s membrane opening and lamina terminalis fenestration postoperatively and at the three-month follow-up.

## Conclusions

Cisternotomy is a safe microsurgical procedure that can effectively decrease ICP and relax the brain. Here, we present a case of IIH treated with cisternotomy, Liliequist’s membrane opening, and lamina terminalis fenestration. This effect can be explained by the recently discovered glymphatic system. Cisternotomy with lamina terminalis fenestration and Liliequist’s membrane opening presents a potential novel surgical option for patients with IIH. Larger multicenter randomized trials are needed to establish the effectiveness of cisternotomy in the management of IIH.
